# Drinking Water Nitrate and Human Health: An Updated Review

**DOI:** 10.3390/ijerph15071557

**Published:** 2018-07-23

**Authors:** Mary H. Ward, Rena R. Jones, Jean D. Brender, Theo M. de Kok, Peter J. Weyer, Bernard T. Nolan, Cristina M. Villanueva, Simone G. van Breda

**Affiliations:** 1 Occupational and Environmental Epidemiology Branch, Division of Cancer Epidemiology and Genetics, National Cancer Institute, 9609 Medical Center Dr. Room 6E138, Rockville, MD 20850, USA; rena.jones@nih.gov; 2 Department of Epidemiology and Biostatistics, Texas A&M University, School of Public Health, College Station, TX 77843, USA; jdbrender@sph.tamhsc.edu; 3 Department of Toxicogenomics, GROW-school for Oncology and Developmental Biology, Maastricht University Medical Center, P.O Box 616, 6200 MD Maastricht, The Netherlands; t.dekok@maastrichtuniversity.nl (T.M.d.K.); s.vanbreda@maastrichtuniversity.nl (S.G.v.B.); 4 The Center for Health Effects of Environmental Contamination, The University of Iowa, 455 Van Allen Hall, Iowa City, IA 52242, USA; peter-weyer@uiowa.edu; 5 U.S. Geological Survey, Water Mission Area, National Water Quality Program, 12201 Sunrise Valley Drive, Reston, VA 20192, USA; btnolan@usgs.gov; 6 ISGlobal, 08003 Barcelona, Spain; cvillanueva@isiglobal.org; 7 IMIM (Hospital del Mar Medical Research Institute), 08003 Barcelona, Spain; 8 Department of Experimental and Health Sciences, Universitat Pompeu Fabra (UPF), 08003 Barcelona, Spain; 9 CIBER Epidemiología y Salud Pública (CIBERESP), 28029 Madrid, Spain

**Keywords:** drinking water, nitrate, cancer, adverse reproductive outcomes, methemoglobinemia, thyroid disease, endogenous nitrosation, *N*-nitroso compounds

## Abstract

Nitrate levels in our water resources have increased in many areas of the world largely due to applications of inorganic fertilizer and animal manure in agricultural areas. The regulatory limit for nitrate in public drinking water supplies was set to protect against infant methemoglobinemia, but other health effects were not considered. Risk of specific cancers and birth defects may be increased when nitrate is ingested under conditions that increase formation of *N*-nitroso compounds. We previously reviewed epidemiologic studies before 2005 of nitrate intake from drinking water and cancer, adverse reproductive outcomes and other health effects. Since that review, more than 30 epidemiologic studies have evaluated drinking water nitrate and these outcomes. The most common endpoints studied were colorectal cancer, bladder, and breast cancer (three studies each), and thyroid disease (four studies). Considering all studies, the strongest evidence for a relationship between drinking water nitrate ingestion and adverse health outcomes (besides methemoglobinemia) is for colorectal cancer, thyroid disease, and neural tube defects. Many studies observed increased risk with ingestion of water nitrate levels that were below regulatory limits. Future studies of these and other health outcomes should include improved exposure assessment and accurate characterization of individual factors that affect endogenous nitrosation.

## 1. Introduction

Since the mid-1920s, humans have doubled the natural rate at which nitrogen is deposited onto land through the production and application of nitrogen fertilizers (inorganic and manure), the combustion of fossil fuels, and replacement of natural vegetation with nitrogen-fixing crops such as soybeans [1,2]. The major anthropogenic source of nitrogen in the environment is nitrogen fertilizer, the application of which increased exponentially after the development of the Haber–Bosch process in the 1920s. Most synthetic fertilizer applications to agricultural land occurred after 1980 [3]. Since approximately half of all applied nitrogen drains from agricultural fields to contaminate surface and groundwater, nitrate concentrations in our water resources have also increased [1].

The maximum contaminant level (MCL) for nitrate in public drinking water supplies in the United States (U.S.) is 10 mg/L as nitrate-nitrogen (NO_3_-N). This concentration is approximately equivalent to the World Health Organization (WHO) guideline of 50 mg/L as NO_3_ or 11.3 mg/L NO_3_-N (multiply NO_3_ mg/L by 0.2258). The MCL was set to protect against infant methemoglobinemia; however other health effects including cancer and adverse reproductive outcomes were not considered [4]. Through endogenous nitrosation, nitrate is a precursor in the formation of *N*-nitroso compounds (NOC); most NOC are carcinogens and teratogens. Thus, exposure to NOC formed after ingestion of nitrate from drinking water and dietary sources may result in cancer, birth defects, or other adverse health effects. Nitrate is found in many foods, with the highest levels occurring in some green leafy and root vegetables [5,6]. Average daily intakes from food are in the range of 30–130 mg/day as NO_3_ (7–29 mg/day NO_3_-N) [5]. Because NOC formation is inhibited by ascorbic acid, polyphenols, and other compounds present at high levels in most vegetables, dietary nitrate intake may not result in substantial endogenous NOC formation [5,7].

Studies of health effects related to nitrate exposure from drinking water were previously reviewed through early 2004 [8]. Further, an International Agency for Research on Cancer (IARC) Working Group reviewed human, animal, and mechanistic studies of cancer through mid-2006 and concluded that ingested nitrate and nitrite, under conditions that result in endogenous nitrosation, are probably carcinogenic [5]. Here, our objective is to provide updated information on human exposure and to review mechanistic and health effects studies since 2004. We summarize how the additional studies contribute to the overall evidence for health effects and we discuss what future research may be most informative.

## 2. Drinking Water Nitrate Exposures in the United States and Europe

**Figure 1:**
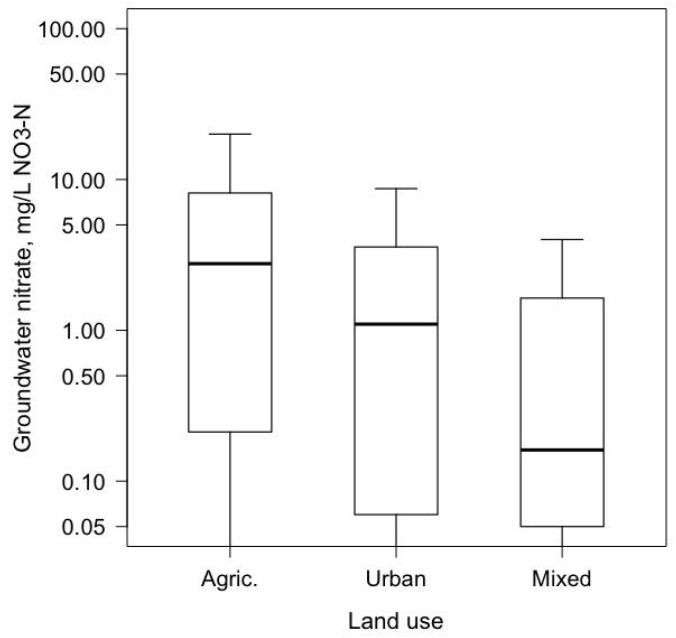
Boxplots of nitrate concentrations in shallow groundwater beneath agricultural and urban land uses, and at depths of private and public drinking water supplies beneath mixed land use. The number of sampled wells were 1573 (agricultural land), 1054 (urban), and 3417 (mixed). The agricultural and urban wells were sampled to assess land use effects, whereas the mixed category wells were sampled at depths of private and public supplies. Median depths of wells in the agricultural, urban, and mixed categories were 34, 32, and 200 feet, respectively. The height of the upper bar is 1.5 times the length of the box, and the lower bound was truncated at the nitrate detection limit of 0.05 mg/L NO_3_-N.

Approximately 45 million people in the U.S. (about 14% of the population) had self-supplied water at their residence in 2010 [9]. Almost all (98%) were private wells, which are not regulated by the U.S. Environmental Protection Agency (EPA). The rest of the population was served by public water supplies, which use groundwater, surface water, or both. The U.S. Geological Survey’s National Water Quality Assessment (USGS-NAWQA) Project [10] sampled principal groundwater aquifers used as U.S. public and private drinking water supplies in 1988–2015. Nitrate levels in groundwater under agricultural land were about three times the national background level of 1 mg/L NO_3_-N (Figure 1) [11]. The mixed land use category mostly had nitrate concentrations below background levels reflecting levels in deeper private and public water supply wells. Based on the NAWQA study, it was estimated that 2% of public-supply wells and 6% of private wells exceeded the MCL; whereas, in agricultural areas, 21% of private wells exceeded the MCL [10]. The USGS-NAWQA study also revealed significant decadal-scale changes in groundwater nitrate concentrations among wells sampled first in 1988–2000 and again in 2001–2010 for agricultural, urban, and mixed land uses [12]. More sampling networks had increases in median nitrate concentration than had decreases.

A study of U.S. public water supplies (PWS) using data from EPA’s Safe Drinking Water Information System estimated that the percentage of PWS violating the MCL increased from 0.28 to 0.42% during 1994–2009; most increases were for small to medium PWS (<10,000 population served) using groundwater [13]. As a result of increasing nitrate levels, some PWS have incurred expensive upgrades to their treatment systems to comply with the regulatory level [14,15,16].

In Europe, the Nitrates Directive was set in 1991 [17,18] to reduce or prevent nitrate pollution from agriculture. Areas most affected by nitrate pollution are designated as ‘nitrate vulnerable zones’ and are subject to mandatory Codes of Good Agricultural Practice [18]. The results of compliance with this directive have been reflected in the time trends of nitrate in some countries. For example, nitrate levels in groundwater in Denmark increased in 1950–1980 and decreased since the 1990s [19]. Average nitrate levels in groundwater in most other European countries have been stable at around 17.5 mg/L NO_3_ (4 mg/L NO_3_-N) across Europe over a 20-year period (1992–2012), with some differences between countries both in trends and concentrations. Average concentrations are lowest in Finland (around 1 mg/L NO_3_ in 1992–2012) and highest in Malta (58.1 mg/L in 2000–2012) [20]. Average annual nitrate concentrations at river monitoring stations in Europe showed a steady decline from 2.7 NO_3_-N in 1992 to 2.1 mg/L in 2012 [20], with the lowest average levels in Norway (0.2 mg/L NO_3_-N in 2012) and highest in Greece (6.6 mg/L NO_3_-N in 2012).

Levels in finished public drinking water have been published only for a few European countries. Trends of nitrate in drinking water supplies from 1976 to 2012 in Denmark showed a decline in public supplies but not in private wells [21]. In Spain, median concentrations were 3.5 mg/L NO_3_ (range: 0.4−66.8) in 108 municipalities in 2012 [22], and 4.2 mg/L (range: <1−29) in 11 provinces in 2010 [23]. Levels in other countries included a median of 0.18 mg/L (range: <0.02−7.9) in Iceland in 2001−2012 [24], a mean of 16.1 mg/L (range: 0.05−296 mg/L) in Sicily, Italy in 2004−2005 [25] and a range from undetected to 63.3 mg/L in Deux-Sèvres, France in in 2005−2009 [26]. 

Nitrate levels in bottled water have been measured in a few areas of the EU and the U.S. and have been found to be below the MCL. In Sicily, the mean level was 15.2 mg/L NO_3_(range: 1.2−31.8 mg/L) in 16 brands [25] and in Spain, the median level was 5.2 mg/L NO_3_ (range: <1.0−29.0 mg/L) in 9 brands [23]. In the U.S., a survey of bottle water sold in 42 Iowa and 32 Texas communities found varying but generally low nitrate levels. Nitrate concentrations ranged from below the limit of detection (0.1 mg/L NO_3_-N) to 4.9 mg/L NO_3_-N for U.S. domestic spring water purchased in Texas.

There are few published studies of nitrate concentrations in drinking water outside the U.S. and Europe. Nitrate concentrations in groundwater were reported for Morocco, Niger, Nigeria, Senegal, India-Pakistan, Japan, Lebanon, Philippines and Turkey with maximum levels in Senegal (median 42.9 mg/L NO_3_-N) [5]. In India, nitrate in drinking water supplies is particularly high in rural areas, where average levels have been reported to be 45.7 mg/L NO_3_ [27,28] and 66.6 mg/L NO_3_ [28]; maximum levels in drinking water exceeded 100 mg/L NO_3_ in several regions [27,29]. Extremely high levels of nitrate have been reported in The Gaza Strip, where nitrate reached concentrations of 500 mg/L NO_3_ in some areas, and more than 50% of public-supply wells had nitrate concentrations above 45 mg/L NO_3_ [30].

## 3. Exposure Assessment in Epidemiologic Studies

With the implementation of the Safe Drinking Water Act in 1974, more than 40 years of monitoring data for public water supplies in the U.S. provide a framework of measurements to support exposure assessments. Historical data for Europe are more limited, but a quadrennial nitrate reporting requirement was implemented as part of the EU Nitrates Directive [17,18]. In the U.S., the frequency of sampling for nitrate in community water systems is stipulated by their sources (ground versus surface waters) and whether concentrations are below the MCL, and historically, by the size of the population served and vulnerability to nitrate contamination. Therefore, the exposure assessment for study participants who report using a public drinking water source may be based on a variable number of measurements, raising concerns about exposure misclassification. In a study of bladder cancer risk in Iowa, associations were stronger in sensitivity analyses based on more comprehensive measurement data [31]. Other studies have restricted analyses to subgroups with more complete or recent measurements [32,33,34,35], with implications for study power and possible selection biases. Sampling frequency also limits the extent to which temporal variation in exposure can be represented within a study population, such as the monthly or trimester-based estimates of exposure most relevant for etiologic investigations of adverse reproductive outcomes. In Denmark, limited seasonal variation in nitrate monitoring data suggested these data would sufficiently capture temporal variation for long-term exposure estimates [36]. Studies have often combined regulatory measurements with questionnaire and ancillary data to better characterize individual variation in nitrate exposure, such as to capture changes in water supply characteristics over time or a participant’s duration at a drinking water source [31,33,37,38]. Most case-control studies of drinking water nitrate and cancer obtained lifetime residence and drinking water source histories, whereas cohort studies typically have collected only the current water source. Many studies lacked information about study participants’ water consumption, which may be an important determinant of exposure to drinking water contaminants [39].

Due to sparse measurement data, exposures for individuals served by private wells are more difficult to estimate than exposures for those on public water supplies. However, advances in geographic-based modeling efforts that incorporate available measurements, nitrogen inputs, aquifer characteristics, and other data hold promise for this purpose. These models include predictor variables describing land use, nitrogen inputs (fertilizer applications, animal feeding operations), soils, geology, climate, management practices, and other factors at the scale of interest. Nolan and Hitt [40] and Messier et al. [41] used nonlinear regression models with terms representing nitrogen inputs at the land surface, transport in soils and groundwater, and nitrate removal by processes such as denitrification, to predict groundwater nitrate concentration at the national scale and for North Carolina, respectively. Predictor variables in the models included N fertilizer and manure, agricultural or forested land use, soils, and, in Nolan and Hitt [40], water-use practices and major geology. Nolan and Hitt [40] reported a training R^2^ values of 0.77 for a model of groundwater used mainly for private supplies and Messier, Kane, Bolich and Serre [41] reported a cross-validation testing R^2^ value of 0.33 for a point-level private well model. These and earlier regression approaches for groundwater nitrate [42,43,44,45,46] relied on predictor variables describing surficial soils and activities at the land surface, because conditions at depth in the aquifer typically are unknown. Redox conditions in the aquifer and the time since water entered the subsurface (i.e., groundwater age) are two of the most important factors affecting groundwater nitrate, but redox constituents typically are not analyzed, and age is difficult to measure. Even if a well has sufficient data to estimate these conditions, the data must be available for all wells in order to predict water quality in unsampled areas. In most of the above studies, well depth was used as a proxy for age and redox and set to average private or public-supply well depth for prediction.

Recent advances in groundwater nitrate exposure modeling have involved machine-learning methods such as random forest (RF) and boosted regression trees (BRT), along with improved characterization of aquifer conditions at the depth of the well screen (the perforated portion of the well where groundwater intake occurs). Tree-based models do not require data transformation, can fit nonlinear relations, and automatically incorporate interactions among predictors [47]. Wheeler et al. [48] used RF to estimate private well nitrate levels in Iowa. In addition to land use and soil variables, predictor variables included aquifer characteristics at the depth of the well screen, such as total thickness of fine-grained glacial deposits above the well screen, average and minimum thicknesses of glacial deposits near sampled wells, and horizontal and vertical hydraulic conductivities near the wells. Well depth, landscape features, nitrogen sources, and aquifer characteristics ranked highly in the final model, which explained 77% and 38% of the variation in training and hold-out nitrate data, respectively.

Ransom et al. [49] used BRT to predict nitrate concentration at the depths of private and public-supply wells for the Central Valley, California. The model used as input estimates of groundwater age at the depth of the well screen (from MODFLOW/MODPATH models) and depth-related reducing conditions in the groundwater. These estimates were generated by separate models and were available throughout the aquifer. Other MODFLOW-based predictor variables comprised depth to groundwater, and vertical water fluxes and the percent coarse material in the uppermost part of the aquifer where groundwater flow was simulated by MODFLOW. Redox variables were top-ranked in the final BRT model, which also included land use-based N leaching flux, precipitation, soil characteristics, and the MODFLOW-based variables described above. The final model retained 25 of an initial 145 predictor variables considered, had training and hold-out R^2^ values of 0.83 and 0.44 respectively, and was used to produce a 3D visualization of nitrate in the aquifer. These studies show that modeling advances and improved characterization of aquifer conditions at depth are increasing our ability to predict nitrate exposure from drinking water supplied by private wells.

## 4. Nitrate Intake and Endogenous Formation of *N*-Nitroso Compounds

Drinking water nitrate is readily absorbed in the upper gastrointestinal tract and distributed in the human body. When it reaches the salivary glands, it is actively transported from blood into saliva and levels may be up to 20 times higher than in the plasma [50,51,52,53]. In the oral cavity 6–7% of the total nitrate can be reduced to nitrite, predominantly by nitrate-reducing bacteria [52,54,55]. The secreted nitrate as well as the nitrite generated in the oral cavity re-enter the gastrointestinal tract when swallowed.

Under acidic conditions in the stomach, nitrite can be protonated to nitrous acid (HNO_2_), and subsequently yield dinitrogen trioxide (N_2_O_3_), nitric oxide (NO), and nitrogen dioxide (NO_2_). Since the discovery of endogenous NO formation, it has become clear that NO is involved in a wide range of NO-mediated physiological effects. These comprise the regulation of blood pressure and blood flow by mediating vasodilation [56,57,58], the maintenance of blood vessel tonus [59], the inhibition of platelet adhesion and aggregation [60,61], modulation of mitochondrial function [62] and several other processes [63,64,65,66].

On the other hand, various nitrate and nitrite derived metabolites such as nitrous acid (HNO_2_) are powerful nitrosating agents and known to drive the formation of NOC, which are suggested to be the causal agents in many of the nitrate-associated adverse health outcomes. NOC comprise *N*-nitrosamines and *N*-nitrosamides, and may be formed when nitrosating agents encounter *N*-nitrosatable amino acids, which are also from dietary origin. The nitrosation process depends on the reaction mechanisms involved, on the concentration of the compounds involved, the pH of the reaction environment, and further modifying factors, including the presence of catalysts or inhibitors of *N*-nitrosation [66,67,68,69].

Endogenous nitrosation can also be inhibited, for instance by dietary compounds like vitamin C, which has the capacity to reduce HNO_2_ to NO; and alpha-tocopherol or polyphenols, which can reduce nitrite to NO [54,70,71,72]. Inhibitory effects on nitrosation have also been described for dietary flavonoids such as quercetin, ferulic and caffeic acid, betel nut extracts, garlic, coffee, and green tea polyphenols [73,74]. Earlier studies showed that the intake of 250 mg or 1 g ascorbic acid per day substantially inhibited *N*-nitrosodimethylamine (NDMA) excretion in 25 women consuming a fish meal rich in amines (nitrosatable precursors) for seven days, in combination with drinking water containing nitrate at the acceptable daily intake (ADI) [75]. In addition, strawberries, garlic juice, and kale juice were shown to inhibit NDMA excretion in humans [76]. The effect of these fruits and vegetables is unlikely to be due solely to ascorbic acid. Using the *N*-nitrosoproline (NPRO) test, Helser et al. [77] found that ascorbic acid only inhibited nitrosamine formation by 24% compared with 41–63% following ingestion of juices (100 mL) made of green pepper, pineapple, strawberry or carrot containing an equal total amount of ascorbic acid.

The protective potential of such dietary inhibitors depends not only on the reaction rates of *N*-nitrosatable precursors and nitrosation inhibitors, but also on their biokinetics, since an effective inhibitor needs to follow gastrointestinal circulation kinetics similar to nitrate [78]. It has been argued that consumption of some vegetables with high nitrate content, can at least partially inhibit the formation of NOC [79,80,81]. This might apply for green leafy vegetables such as spinach and rocket salad, celery or kale [77] as well as other vegetables rich in both nitrate and natural nitrosation inhibitors. Preliminary data show that daily consumption of one bottle of beetroot juice containing 400 mg nitrate (the minimal amount advised for athletes to increase their sports performances) for one day and seven days by 29 young individuals results in an increased urinary excretion of apparent total nitroso compounds (ATNC), an effect that can only be partially inhibited by vitamin C supplements (1 g per day) [82].

Also, the amount of nitrosatable precursors is a key factor in the formation of NOC. Dietary intakes of red and processed meat are of particular importance [83,84,85,86,87] as increased consumption of red meat (600 vs. 60 g/day), but not white meat, was found to cause a three-fold increase in fecal NOC levels [85]. It was demonstrated that heme iron stimulated endogenous nitrosation [84], thereby providing a possible explanation for the differences in colon cancer risk between red and white meat consumption [88]. The link between meat consumption and colon cancer risk is even stronger for nitrite-preserved processed meat than for fresh meat leading an IARC review to conclude that processed meat is carcinogenic to humans [89].

In a human feeding study [90], the replacement of nitrite in processed meat products by natural antioxidants and the impact of drinking water nitrate ingestion is being evaluated in relation to fecal excretion of NOC, accounting for intakes of meat and dietary vitamin C. A pilot study demonstrated that fecal excretion of ATNC increased after participants switched from ingesting drinking water with low nitrate levels to drinking water with nitrate levels at the acceptable daily intake level of 3.7 mg/kg. The 20 volunteers were assigned to a group consuming either 3.75 g/kg body weight (maximum 300 g per day) red processed meat or fresh (unprocessed) white meat. Comparison of the two dietary groups showed that the most pronounced effect of drinking water nitrate was observed in the red processed meat group. No inhibitory effect of vitamin C intake on ATNC levels in feces was found (unpublished results).

## 5. Methemoglobinemia

The physiologic processes that can lead to methemoglobinemia in infants under six months of age have been described in detail previously [8,91]. Ingested nitrate is reduced to nitrite by bacteria in the mouth and in the infant stomach, which is less acidic than adults. Nitrite binds to hemoglobin to form methemoglobin, which interferes with the oxygen carrying capacity of the blood. Methemoglobinemia is a life-threatening condition that occurs when methemoglobin levels exceed about 10% [8,91]. Risk factors for infant methemoglobinemia include formula made with water containing high nitrate levels, foods and medications that have high nitrate levels [91,92], and enteric infections [93]. Methemoglobinemia related to high nitrate levels in drinking water used to make infant formula was first reported in 1945 [94]. The U.S. EPA limit of 10 mg/L NO_3_-N was set as about one-half the level at which there were no observed cases [95]. The most recent U.S. cases related to nitrate in drinking water were reported by Knobeloch and colleagues in the late 1990s in Wisconsin [96] and were not described in our prior review. Nitrate concentrations in the private wells were about two-times the MCL and bacterial contamination was not a factor. They also summarize another U.S. case in 1999 related to nitrate contamination of a private well and six infant deaths attributed to methemoglobinemia in the U.S. between 1979–1999 only one of which was reported in the literature [96,97]. High incidence of infant methemoglobinemia in eastern Europe has also been described previously [98,99]. A 2002 WHO report on water and health [100] noted that there were 41 cases in Hungary annually, 2913 cases in Romania from 1985–1996 and 46 cases in Albania in 1996.

Results of several epidemiologic studies conducted before 2005 that examined the relationship between nitrate in drinking water and levels of methemoglobin or methemoglobinemia in infants have been described previously [8]. Briefly, nitrate levels >10 mg/L NO_3_-N were usually associated with increased methemoglobin levels but clinical methemoglobinemia was not always present. Since our last review, a cross-sectional study conducted in Gaza found elevated methemoglobin levels in infants on supplemental feeding with formula made from well water in an area with the highest mean nitrate concentration of 195 mg/L NO_3_ (range: 18–440) compared to an area with lower nitrate concentration (mean: 119 mg/L NO_3_; range 18–244) [101]. A cross-sectional study in Morocco found a 22% increased risk of methemoglobinemia in infants in an area with drinking water nitrate >50 mg/L (>11 as NO_3_-N) compared to infants in an area with nitrate levels <50 mg/L nitrate [102]. A retrospective cohort study in Iowa of persons (aged 1–60 years) consuming private well water with nitrate levels <10 mg/L NO_3_-N found a positive relationship between methemoglobin levels in the blood and the amount of nitrate ingestion [103]. Among pregnant women in rural Minnesota with drinking water supplies that were mostly ≤3 mg/L NO_3_-N, there was no relationship between water nitrate intake and women’s methemoglobin levels around 36 weeks’ gestation [104].

## 6. Adverse Pregnancy Outcomes

Maternal drinking water nitrate intake during pregnancy has been investigated as a risk factor for a range of pregnancy outcomes, including spontaneous abortion, fetal deaths, prematurity, intrauterine growth retardation, low birth weight, congenital malformations, and neonatal deaths. The relation between drinking water nitrate and congenital malformations in offspring has been the most extensively studied, most likely because of the availability of birth defect surveillance systems around the world.

Our earlier review focused on studies of drinking water nitrate and adverse pregnancy outcomes published before 2005 [8]. In that review, we cited several studies on the relation between maternal exposure to drinking water nitrate and spontaneous abortion including a cluster investigation that suggested a positive association [105] and a case-control study that found no association [106]. These studies were published over 20 years ago. In the present review, we were unable to identify any recently published studies on this outcome. In Table 1, we describe the findings of studies published since 2004 on the relation between drinking water nitrate and prematurity, low birthweight, and congenital malformations. We report results for nitrate in the units (mg/L NO_3_ or NO_3_-N) that were reported in the publications. In a historic cohort study conducted in the Deux-Sèvres district (France), Migeot et al. [26] linked maternal addresses from birth records to community water system measurements of nitrate, atrazine, and other pesticides. Exposure to the second tertile of nitrate (14–27 mg/L NO_3_) without detectable atrazine metabolites was associated with small-for-gestational age births (Odds Ratio (OR) 1.74, 95% CI 1.1, 2.8), but without a monotonic increase in risk with exposures. There was no association with nitrate among those with atrazine detected in their drinking water supplies. Within the same cohort, Albouy-Llaty and colleagues did not observe any association between higher water nitrate concentrations (with or without the presence of atrazine) and preterm birth [107]. 

Stayner and colleagues also investigated the relation between atrazine and nitrate in drinking water and rates of low birth weight and preterm birth in 46 counties in four Midwestern U.S. states that were required by EPA to measure nitrate and atrazine monthly due to prior atrazine MCL violations [108]. The investigators developed county-level population-weighted metrics of average monthly nitrate concentrations in public drinking water supplies. When analyses were restricted to counties with less than 20% private well usage (to reduce misclassification due to unknown nitrate levels), average nitrate concentrations during the pregnancy were associated with increased rates of very low birth weight (<1.5 kg Rate Ratio (RR)_per 1 ppm_ = 1.17, 95% CI 1.08, 1.25) and very preterm births (<32 weeks RR_per 1 ppm_ = 1.08, 95% CI 1.02, 1.15) but not with low birth weight or preterm birth overall.

In record-based prevalence study in Perth Australia, Joyce et al. mapped births to their water distribution zone and noted positive associations between increasing tertiles of nitrate levels and prevalence of term premature rupture of membranes (PROM) adjusted for smoking and socioeconomic status [109]. Nitrate concentrations were low; the upper tertile cut point was 0.350 mg/L and the maximum concentration was 1.80 mg/L NO_3_-N. Preterm PROM was not associated with nitrate concentrations.

Among studies of drinking water nitrate and congenital malformations, few before 2005 included birth defects other than central nervous system defects [8]. More recently, Mattix et al. [110] noted higher rates of abdominal wall defects (AWD) in Indiana compared to U.S. rates for specific years during the period 1990–2002. They observed a positive correlation between monthly AWD rates and monthly atrazine concentrations in surface waters but no correlation with nitrate levels. Water quality data were obtained from the USGS-NAWQA project that monitors agricultural chemicals in streams and shallow groundwater that are mostly not used as drinking water sources. A case-control study of gastroschisis (one of the two major types of AWD), in Washington State [111] also used USGS-NAWQA measurements of nitrate and pesticides in surface water and determined the distance between maternal residences (zip code centroids) and the closest monitoring site with concentrations above the MCL for nitrate, nitrite, and atrazine. Gastrochisis was not associated with maternal proximity to surface water above the MCL for nitrate (>10 mg/L NO_3_-N) or nitrite (>1 mg/L NO_2_-N) but there was a positive relationship with proximity to sites with atrazine concentrations above the MCL. In a USA-wide study, Winchester et al. [112] linked the USGS-NAWQA monthly surface water nitrate and pesticide concentrations computed for the month of the last menstrual period with monthly rates of 22 types of birth defects in 1996–2002. Rates of birth defects among women who were estimated to have conceived during April through July were higher than rates among women conceiving in other months. In multivariable models that included nitrate, atrazine, and other pesticides, atrazine (but not nitrate or other pesticides) was associated with several types of anomalies. Nitrate was associated with birth defects in the category of “other congenital anomalies” (OR 1.18, 95% CI 1.14, 1.21); the authors did not specify what defects were included in this category. None of these three studies included local or regional data to support the assumption that surface water nitrate and pesticide concentrations correlated with drinking water exposures to these contaminants.

Using a more refined exposure assessment than the aforementioned studies, Holtby et al. [113] conducted a case-control study of congenital anomalies in an agricultural county in Nova Scotia, Canada. They linked maternal addresses at delivery to municipal water supply median nitrate concentrations and used kriging of monthly measurements from a network of 140 private wells to estimate drinking water nitrate concentrations in private wells. They observed no associations between drinking water nitrate and all birth defects combined for conceptions during 1987–1997. However, the prevalence of all birth defects occurring during 1998–2006 was associated with drinking water nitrate concentrations of 1–5.56 mg/L NO_3_-N (OR 2.44, 95% CI 1.05, 5.66) and ≥5.56 mg/L (OR 2.25, 95% CI 0.92, 5.52).

None of the studies of congenital anomalies accounted for maternal consumption of bottled water or the quantity of water consumed during the first trimester, the most critical period of organ/structural morphogenesis. Attempting to overcome some of these limitations, Brender, Weyer, and colleagues [38,114] conducted a population-based, case-control study in the states of Iowa and Texas where they: (1) linked maternal addresses during the first trimester to public water utilities and respective nitrate measurements; (2) estimated nitrate intake from bottled water based on a survey of products consumed and measurement of nitrate in the major products; (3) predicted drinking water nitrate from private wells through modeling (Texas only); and (4) estimated daily nitrate ingestion from women’s drinking water sources and daily consumption of water. The study populations were participants of the U.S. National Birth Defects Prevention Study [115]. Compared to the lowest tertile of nitrate ingestion from drinking water (<0.91 mg/day NO_3_), mothers of babies with spina bifida were twice as likely (95% CI 1.3, 3.2) to ingest ≥5 mg/day NO_3_ from drinking water than control mothers. Mothers of babies with limb deficiencies, cleft palate, and cleft lip were, respectively, 1.8 (95% CI 1.1, 3.1), 1.9 (95% CI 1.2, 3.1), and 1.8 (95% CI 1.1, 3.1) times more likely to ingest ≥5.4 mg/day of water NO_3_ than controls. Women were also classified by their nitrosatable drug exposure during the first trimester [116] and by their daily nitrate and nitrite intake based on a food frequency questionnaire [117]. Higher ingestion of drinking water nitrate did not strengthen associations between maternal nitrosatable drug exposure and birth defects in offspring [38]. However, a pattern was observed of stronger associations between nitrosatable drug exposure and selected birth defects for women in the upper two tertiles of total nitrite ingestion that included contributions from drinking water nitrate and dietary intakes of nitrate and nitrite compared to women in the lowest tertile. Higher intake of food nitrate/nitrite was found to also modify the associations of nitrosatable drug exposure and birth defects in this study [118,119] as well as in an earlier study of neural tube defects conducted in south Texas [120]. Multiplicative interactions were observed between higher food nitrate/nitrite and nitrosatable drug exposures for conotruncal heart, limb deficiency, and oral cleft defects [118].

In summary, five out of six studies, conducted since the 1980s of drinking water nitrate and central nervous system defects, found positive associations between higher drinking water nitrate exposure during pregnancy and neural tube defects or central nervous system defects combined [38,120,121,122,123]. The sixth study, which did not find a relationship, did not include measures of association, but compared average drinking water nitrate concentrations between mothers with and without neural tube defect-affected births, which were comparable [124].

## 7. Cancer

Most early epidemiologic studies of cancer were ecologic studies of stomach cancer mortality that used exposure estimates concurrent with the time of death. Results were mixed, with some studies showing positive associations, many showing no association, and a few showing inverse associations. The results of ecologic studies through 1995 were reviewed by Cantor [125]. Our previous review included ecologic studies of the brain, esophagus, stomach, kidney, ovary, and non-Hodgkin lymphoma (NHL) published between 1999 and 2003 that were largely null [8]. We did not include ecologic studies or mortality case-control studies in this review due to the limitations of these study designs, especially their inability to assess individual-level exposure and dietary factors that influence the endogenous formation of NOC.

Since our review of drinking water nitrate and health in 2005 [8], eight case-control studies and eight analyses in three cohorts have evaluated historical nitrate levels in PWS in relation to several cancers. Nitrate levels were largely below 10 mg/L NO_3_-N. Most of these studies evaluated potential confounders and factors affecting nitrosation. Table 2 shows the study designs and results of studies published from 2005 through 2018, including findings from periodic follow-ups of a cohort study of postmenopausal women in Iowa (USA) [31,37,126,127,128,129]. In the first analysis of drinking water nitrate in the Iowa cohort with follow-up through 1998, Weyer and colleagues [130] reported that ovarian and bladder cancers were positively associated with the long-term average PWS nitrate levels prior to enrollment (highest quartile average 1955–1988: >2.46 mg/L NO_3_-N). They observed inverse associations for uterine and rectal cancer, but no associations with cancers of the breast, colon, rectum, pancreas, kidney, lung, melanoma, non-Hodgkin lymphoma (NHL), or leukemia. Analyses of PWS nitrate concentrations and cancers of the thyroid, breast, ovary, bladder, and kidney were published after additional follow-up of the cohort. The exposure assessment was improved by: (a) the computation of average nitrate levels and years of exposure at or above 5 mg/L NO_3_-N, based on time in residence (vs. one long-term PWS average nitrate estimate used by Weyer and colleagues); and (b) by estimation of total trihalomethanes (TTHM) and dietary nitrite intake.

Thyroid cancer was evaluated for the first time after follow-up of the cohort through 2004. A total of 40 cases were identified [37]. Among women with >10 years on PWS with levels exceeding 5 mg/L NO_3_-N for five years or more, thyroid cancer risk was 2.6 times higher than that of women whose supplies never exceeded 5 mg/L. With follow-up through 2010, the risk of ovarian cancer remained increased among women in the highest quartile of average nitrate in PWS [129]. Ovarian cancer risk among private well users was also elevated compared to the lowest PWS nitrate quartile. Associations were stronger when vitamin C intake was below median levels with a significant interaction for users of private wells. Overall, breast cancer risk was not associated with water nitrate levels with follow-up through 2008 [128]. Among women with folate intake ≥ 400 μg/day, risk was increased for those in the highest average nitrate quintile (Hazard Ratio (HR) = 1.40; 95% CI: = 1.05–1.87) and among private well users (HR = 1.38; 95% CI: = 1.05–1.82), compared to those with the lowest average nitrate quintile. There was no association with nitrate exposure among women with lower folate intake. With follow-up through 2010, there were 130 bladder cancer cases among women who had used PWS >10 years. Risk remained elevated among women with the highest average nitrate levels and was 1.6 times higher among women whose drinking water concentration exceeded 5 mg/L NO_3_-N for at least four years [31]. Risk estimates were not changed by adjustment for TTHM, which are suspected bladder cancer risk factors. Smoking, but not vitamin C intake, modified the association with nitrate in water; increased risk was apparent only in current smokers (*p*-interaction <0.03). With follow-up through 2010, there were 125 kidney cancer cases among women using PWS; risk was increased among those in the 95th percentile of average nitrate (>5.0 mg/L NO_3_-N) compared with the lowest quartile (HR = 2.2, 95% CI: 1.2–4.2) [127]. There was no positive trend with the average nitrate level and no increased risk for women using private wells, compared to those with low average nitrate in their public supply. An investigation of pancreatic cancer in the same population (follow-up through 2011) found no association with average water nitrate levels in public supplies and no association among women on private wells [126].

In contrast to the positive findings for bladder cancer among the cohort of Iowa women, a cohort study of men and women aged 55–69 in the Netherlands with lower nitrate levels in PWS found no association between water nitrate ingestion (median in top quintile = 2.4 mg/day NO_3_-N) and bladder cancer risk [131]. Dietary intake of vitamins C and E and history of cigarette smoking did not modify the association. A hospital-based case-control study of bladder cancer in multiple areas of Spain [33] assessed lifetime water sources and usual intake of tap water. Nitrate levels in PWS were low, with almost all average levels below 2 mg/L NO_3_-N. Risk of bladder cancer was not associated with the nitrate level in drinking water or with estimated nitrate ingestion from drinking water, and there was no evidence of interaction with factors affecting endogenous nitrosation.

Several case-control studies conducted in the Midwestern U.S. obtained lifetime histories of drinking water sources and estimated exposure for PWS users. In contrast to findings of an increased risk of NHL associated with nitrate levels in Nebraska PWS in an earlier study [132], there was no association with similar concentrations in public water sources in a case-control study of NHL in Iowa [35]. A study of renal cell carcinoma in Iowa [34] found no association with the level of nitrate in PWS, including the number of years that levels exceeded 5 or 10 mg/L NO_3_-N. However, higher nitrate levels in PWS increased risk among subgroups who reported above the median intake of red meat intake or below the median intake of vitamin C (*p*-interaction <0.05). A small case-control study of adenocarcinoma of the stomach and esophagus among men and women in Nebraska [133] estimated nitrate levels among long-term users of PWS and found no association between average nitrate levels and risk.

A case-control study of colorectal cancer among rural women in Wisconsin estimated nitrate levels in private wells using spatial interpolation of nitrate concentrations from a 1994 water quality survey and found increased risk of proximal colon cancer among women estimated to have nitrate levels >10 mg/L NO_3_-N compared to levels < 0.5 mg/L. Risk of distal colon cancer and rectal cancer were not associated with nitrate levels [134]. Water nitrate ingestion from public supplies, bottled water, and private wells and springs over the adult lifetime was estimated in analyses that pooled case-control studies of colorectal cancer in Spain and Italy [135]. Risk of colorectal cancer was increased among those with >2.3 mg/day NO_3_-N (vs. <1.1 mg/day). There were no interactions with red meat, vitamins C and E, and fiber except for a borderline interaction (*p*-interaction = 0.07) for rectum cancer with fiber intake. A small hospital-based case-control study in Indonesia found that drinking water nitrate levels above the WHO standard (>11.3 mg/L as NO_3_-N) was associated with colorectal cancer [136]. A national registry-based cohort study in Denmark [32] evaluated average nitrate concentrations in PWS and private wells in relation to colorectal cancer incidence among those whose 35th birthday occurred during 1978–2011. The average nitrate level was computed over residential water supplies from age 20 to 35. Increased risks for colon and rectum cancer were observed in association with average nitrate levels ≥9.25 mg/L NO_3_ (≥2.1 as NO_3_-N) and ≥3.87 mg/L NO_3_ (>0.87 as NO_3_-N), respectively, with a significant positive trend. Because the study did not interview individuals, it could not evaluate individual-level risk factors that might influence endogenous nitrosation.

A case-control study of breast cancer in Cape Cod, Massachusetts (US) [137] estimated nitrate concentrations in PWS over approximately 20 years as an historical proxy for wastewater contamination and potential exposure to endocrine disruption compounds. Average exposures >1.2 mg/L NO_3_-N (vs. <0.3 mg/L) were not associated with risk. A hospital-based case-control study in Spain found no association between water nitrate ingestion and pre- and post-menopausal breast cancers [138].

Animal studies demonstrate that in utero exposure to nitrosamides can cause brain tumors in the exposed offspring. Water nitrate and nitrite intake during pregnancy was estimated in a multi-center case-control study of childhood brain tumors in five countries based on the maternal residential water source [139]. Results for the California and Washington State sites were reported in our previous review [8,140]. Nitrate/nitrite levels in water supplies were measured using a nitrate test strip method in four countries including these U.S. sites; most of these measurements occurred many years after the pregnancy. Measured nitrate concentrations were not associated with risk of childhood brain tumors. However, higher nitrite levels (>1.5 mg/L NO_2_-N) in the drinking water were associated with increased risk of astrocytomas.

## 8. Thyroid Disease

Animal studies demonstrate that ingestion of nitrate at high doses can competitively inhibit iodine uptake and induce hypertrophy of the thyroid gland [141]. An early study of women in the Netherlands consuming water with nitrate levels at or above the MCL, found increased prevalence of thyroid hypertrophy [142]. Since the last review, five studies have evaluated nitrate ingestion from drinking water (the Iowa cohort study also assessed diet) and prevalence of thyroid disease. A study of school-age children in Slovakia found increased prevalence of subclinical hypothyroidism among children in an area with high nitrate levels (51–274 mg/L NO_3_) in water supplies compared with children ingesting water with nitrate ≤50 mg/L (11 mg/L NO_3_-N). In Bulgarian villages with high nitrate levels (75 mg/L NO_3_) and low nitrate levels (8 mg/L), clinical examinations of the thyroids of pregnant women and school children revealed an approximately four- and three-fold increased prevalence of goiter, respectively, in the high nitrate village [143,144]. The iodine status of the populations in both studies was adequate. Self-reported hypothyroidism and hyperthyroidism among a cohort of post-menopausal women in Iowa was not associated with average nitrate concentrations in PWS [37]. However, dietary nitrate, the predominant source of intake, was associated with increased prevalence of hypothyroidism but not hyperthyroidism. Modeled estimates of nitrate concentrations in private wells among a cohort of Old Order Amish in Pennsylvania (USA) were associated with increased prevalence of subclinical hypothyroidism as determined by thyroid stimulating hormone measurements, among women but not men [145].

## 9. Other Health Effects

Associations between nitrate in drinking water and other non-cancer health effects, including type 1 childhood diabetes (T1D), blood pressure, and acute respiratory tract infections in children were previously reviewed [8]. Since 2004, a small number of studies have contributed additional mixed evidence for these associations. Animal studies indicate that NOC may play a role in the pathology of T1D through damage to pancreatic beta cells [146]. A registry-based study in Finland [147] found a positive trend in T1D incidence with levels of nitrate in drinking water. In contrast, an ecological analysis in Italy showed an inverse correlation with water nitrate levels and T1D rates [148]. A small T1D case-control study in Canada with 57 cases showed no association between T1D and estimated intake of nitrate from drinking water (highest quartile >2.7 mg/day NO_3_-N) [149]. Concentrations of nitrate in drinking water (median ~2.1 mg/L NO_3_-N) were not associated with progression to T1D in a German nested case-control study of islet autoantibody-positive children, who may be at increased risk of the disease [150].

In a prospective, population-based cohort study in Wisconsin (USA), increased incidence of early and late age-related macular degeneration was positively associated with higher nitrate levels (≥5 mg/L vs. <5 mg/L NO_3_-N) in rural private drinking water supplies [151]. The authors suggested several possible mechanisms, including methemoglobin-induced lipid peroxidation in the retina.

Potential benefits of nitrate ingestion include lowering of blood pressure due to production of nitric oxide in the acidic stomach and subsequent vasodilation, antithrombotic, and immunoregulatory effects [152]. Experimental studies in animals and controlled feeding studies in humans have demonstrated mixed evidence of these effects and on other cardiovascular endpoints such as vascular hypertrophy, heart failure, and myocardial infarction (e.g., [152,153,154]). Ingested nitrite from diet has also been associated with increased blood flow in certain parts of the brain [155]. Epidemiologic studies of these effects are limited to estimation of dietary exposures or biomarkers that integrate exposures from nitrate from diet and drinking water. Recent findings in the Framingham Offspring Study suggested that plasma nitrate was associated with increased overall risk of death that attenuated when adjusted for glomerular function (HR: 1.16, 95% CI: 1.0–1.35) but no association was observed for incident cardiovascular disease [156]. No epidemiologic studies have specifically evaluated nitrate ingested from drinking water in relation to these outcomes. Another potential beneficial effect of nitrate is protection against bacterial infections via its reduction to nitrite by enteric bacteria. In an experimental inflammatory bowel disease mouse model, nitrite in drinking water was associated with both preventive and therapeutic effects [157]. However, there is limited epidemiologic evidence for a reduced risk of gastrointestinal disease in populations with high drinking water nitrate intake. One small, cross-sectional study in Iran found no association between nitrate levels in public water supplies with mean levels of ~5.6 mg/L NO_3_-N and gastrointestinal disease [158].

## 10. Discussion

Since our last review of studies through 2004 [8], more than 30 epidemiologic studies have evaluated drinking water nitrate and risk of cancer, adverse reproductive outcomes, or thyroid disease. However, the number of studies of any one outcome was not large and there are still too few studies to allow firm conclusions about risk. The most common endpoints studied were colorectal cancer, bladder, and breast cancer (three studies each) and thyroid disease (four studies). Considering all studies to date, the strongest evidence for a relationship between drinking water nitrate ingestion and adverse health outcomes (besides methemoglobinemia) is for colorectal cancer, thyroid disease, and neural tube defects. Four of the five published studies of colorectal cancer found evidence of an increased risk of colorectal cancer or colon cancer associated with water nitrate levels that were mostly below the respective regulatory limits [32,134,135,159]. In one of the four positive studies [159], increased risk was only observed in subgroups likely to have increased nitrosation. Four of the five studies of thyroid disease found evidence for an increased prevalence of subclinical hypothyroidism with higher ingestion of drinking water nitrate among children, pregnant women, or women only [37,144,145,160]. Positive associations with drinking water nitrate were observed at nitrate concentrations close to or above the MCL. The fifth study, a cohort of post-menopausal women in Iowa, had lower drinking water nitrate exposure but observed a positive association with dietary nitrate [37]. To date, five of six studies of neural tube defects showed increased risk with exposure to drinking water nitrate below the MCL. Thus, the evidence continues to accumulate that higher nitrate intake during the pregnancy is a risk factor for this group of birth defects.

All but one of the 17 cancer studies conducted since 2004 were in the U.S. or Europe, the majority of which were investigations of nitrate in regulated public drinking water. Thyroid cancer was studied for the first time [37] with a positive finding that should be evaluated in future studies. Bladder cancer, a site for which other drinking water contaminants (arsenic, disinfection by-products [DBPs]) are established or suspected risk factors, was not associated with drinking water nitrate in three of the four studies. Most of the cancer studies since 2004 evaluated effect modification by factors known to influence endogenous nitrosation, although few observed evidence for these effects. Several studies of adverse reproductive outcomes since 2004 have indicated a positive association between maternal prenatal exposure to nitrate concentrations below the MCL and low birth weight and small for gestational age births. However, most studies did not account for co-exposure to other water contaminants, nor did they adjust for potential risk factors. The relation between drinking water nitrate and spontaneous abortion continues to be understudied. Few cases of methemoglobinemia, the health concern that lead to the regulation of nitrate in public water supplies, have been reported in the U.S. since the 1990s. However, as described by Knobeloch et al. [96], cases may be underreported and only a small proportion of cases are thoroughly investigated and described in the literature. Based on published reports, [100] areas of the world of particular concern include several eastern European countries, Gaza, and Morocco, where high nitrate concentrations in water supplies have been linked to high levels of methemoglobin in children. Therefore, continued surveillance and education of physicians and parents will be important. Biological plausibility exists for relationships between nitrate ingestion from drinking water and a few other health outcomes including diabetes and beneficial effects on the cardiovascular system, but there have been only a limited number of epidemiologic studies.

Assessment of drinking water nitrate exposures in future studies should be improved by obtaining drinking water sources at home and at work, estimating the amount of water consumed from each source, and collecting information on water filtration systems that may impact exposure. These efforts are important for reducing misclassification of exposure. Since our last review, an additional decade of PWS monitoring data are available in the U.S. and European countries, which has allowed assessment of exposure over a substantial proportion of participants’ lifetimes in recent studies. Future studies should estimate exposure to multiple water contaminants as has been done in recent cancer studies [31,33,127,129]. For instance, nitrate and atrazine frequently occur together in drinking water in agricultural areas [161] and animal studies have found this mixture to be teratogenic [162]. Regulatory monitoring data for pesticides in PWS has been available for over 20 years in the U.S.; therefore, it is now feasible to evaluate co-exposure to these contaminants. Additionally, water supplies in agricultural areas that rely on alluvial aquifers or surface water often have elevated levels of both DBPs and nitrate. Under this exposure scenario, there is the possibility of formation of the nitrogenated DBPs including the carcinogenic NDMA, especially if chloramination treatment is used for disinfection [163,164]. Studies of health effects in countries outside the U.S. and Europe are also needed.

A comprehensive assessment of nitrate and nitrite from drinking water and dietary sources as well as estimation of intakes of antioxidants and other inhibitors of endogenous nitrosation including dietary polyphenols and flavonoids is needed in future studies. Heme iron from red meat, which increases fecal NOC in human feeding studies, should also be assessed as a potential effect modifier of risk from nitrate ingestion. More research is needed on the potential interaction of nitrate ingestion and nitrosatable drugs (those with secondary and tertiary amines or amides). Evidence from several studies of birth defects [38,118,119,120] implicates nitrosatable drug intake during pregnancy as a risk factor for specific congenital anomalies especially in combination with nitrate. Drugs with nitrosatable groups include many over-the-counter and prescription drugs. Future studies with electronic medical records and record-linkage studies in countries like Denmark with national pharmacy data may provide opportunities for evaluation of these exposures.

Populations with the highest exposure to nitrate from their drinking water are those living in agricultural regions, especially those drinking water from shallow wells near nitrogen sources (e.g., crop fields, animal feeding operations). Estimating exposure for private well users is important because it allows assessment of risk over a greater range of nitrate exposures compared to studies focusing solely on populations using PWS. Future health studies should focus on these populations, many of which may have been exposed to elevated nitrate in drinking water from early childhood into adulthood. A major challenge in conducting studies in these regions is the high prevalence of private well use with limited nitrate measurement data for exposure assessment. Recent efforts to model nitrate concentrations in private wells have shown that it is feasible to develop predictive models where sufficient measurement data are available [41,48,49]. However, predictive models from one area are not likely to be directly translatable to other geographic regions with different aquifers, soils, and nitrogen inputs.

Controlled human feeding studies have demonstrated that endogenous nitrosation occurs after ingestion of drinking water with nitrate concentrations above the MCL of 10 mg/L NO_3_-N (~44 mg/L as NO_3_). However, the extent of NOC formation after ingestion of drinking water with nitrate concentrations below the MCL has not been well characterized. Increased risks of specific cancers and central nervous system birth defects in study populations consuming nitrate below the MCL is indirect evidence that nitrate ingestion at these levels may be a risk factor under some conditions. However, confounding by other exposures or risk factors can be difficult to rule out in many studies. Controlled human studies to evaluate endogenous nitrosation at levels below the MCL are needed to understand interindividual variability and factors that affect endogenous nitrosation at drinking water nitrate levels below the MCL.

A key step in the endogenous formation of NOC is the reduction of nitrate, which has been transported from the bloodstream into the saliva, to nitrite by the nitrate-reducing bacteria that are located primarily in the crypts on the back of the tongue [165,166,167]. Tools for measuring bacterial DNA and characterizing the oral microbiome are now available and are currently being incorporated into epidemiologic studies [168,169]. Buccal cell samples that have been collected in epidemiologic studies can be used to characterize the oral microbiome and to determine the relative abundance of the nitrate-reducing bacteria. Studies are needed to characterize the stability of the nitrate-reducing capacity of the oral microbiome over time and to determine factors that may modify this capacity such as diet, oral hygiene, and periodontal disease. Interindividual variability in the oral nitrate-reducing bacteria may play an important role in modifying endogenous NOC formation. The quantification of an individual’s nitrate-reducing bacteria in future epidemiologic studies is likely to improve our ability to classify participants by their intrinsic capacity for endogenous nitrosation.

In addition to characterizing the oral microbiome, future epidemiologic studies should incorporate biomarkers of NOC (e.g., urinary or fecal NOC), markers of genetic damage, and determine genetic variability in NOC metabolism. As many NOC require α-hydroxylation by CYP2E1 for bioactivation and for formation of DNA adducts, it is important to investigate the influence of polymorphisms in the gene encoding for this enzyme. Studies are also needed among populations with medical conditions that increase nitrosation such as patients with inflammatory bowel disease and periodontal disease [8]. Because NOC exposures induce characteristic gene expression profiles [170,171], further studies linking drinking water intake to NOC excretion and gene expression responses are relevant to our understanding of health risks associated with drinking water nitrate. The field of ‘Exposome-research’ [172,173] generates large numbers of genomics profiles in human population studies for which dietary exposures and biobank materials are also available. These studies provide opportunities to measure urinary levels of nitrate and NOC that could be associated with molecular markers of exposure and disease risk.

Nitrate concentrations in global water supplies are likely to increase in the future due to population growth, increases in nitrogen fertilizer use, and increasing intensity and concentration of animal agriculture. Even with increased inputs, mitigation of nitrate concentrations in water resources is possible through local, national, and global efforts. Examples of the latter are the International Nitrogen Initiative [174] and the EU Nitrates Directive [17,18], which aim to quantify human effects on the nitrogen cycle and to validate and promote methods for sustainable nitrogen management. Evidence for the effectiveness of these efforts, which include the identification of vulnerable areas, establishment of codes of good agricultural practices, and national monitoring and reporting are indicated by decreasing trends in groundwater nitrate concentrations in some European countries after the implementation of the EU Nitrates Directive [19]. However, the effect of this initiative was variable across the EU. In the U.S., nitrogen applications to crop fields are not regulated and efforts to reduce nitrogen runoff are voluntary. Although strategies such as appropriate timing of fertilizer applications, diversified crop rotations, planting of cover crops, and reduced tillage can be effective [175], concentrations in U.S. ground and surface water have continued to increase in most areas [10]. Climate change is expected to affect nitrogen in aquatic ecosystems and groundwater through alterations of the hydrological cycle [176]. Climatic factors that affect nitrate in groundwater include the amount, intensity, and timing of precipitation. Increasing rainfall intensity, especially in the winter and spring, can lead to increases in nitrogen runoff from agricultural fields and leaching to groundwater.

## 11. Conclusions

In summary, most adverse health effects related to drinking water nitrate are likely due to a combination of high nitrate ingestion and factors that increase endogenous nitrosation. Some of the recent studies of cancer and some birth defects have been able to identify subgroups of the population likely to have greater potential for endogenous nitrosation. However, direct methods of assessing these individuals are needed. New methods for quantifying the nitrate-reducing bacteria in the oral microbiome and characterizing genetic variation in NOC metabolism hold promise for identifying high risk groups in epidemiologic studies.

To date, the number of well-designed studies of individual health outcomes is still too few to draw firm conclusions about risk from drinking water nitrate ingestion. Additional studies that incorporate improved exposure assessment for populations on PWS, measured or predicted exposure for private well users, quantification of nitrate-reducing bacteria, and estimates of dietary and other factors affecting nitrosation are needed. Studies of colorectal cancer, thyroid disease, and central nervous system birth defects, which show the most consistent associations with water nitrate ingestion, will be particularly useful for clarifying these risks. Future studies of other health effects with more limited evidence of increased risk are also needed including cancers of the thyroid, ovary, and kidney, and the adverse reproductive outcomes of spontaneous abortion, preterm birth, and small for gestational age births.

## Figures and Tables

**Table 1:** Studies of drinking water nitrate ^a^ and adverse pregnancy outcomes published January 2005–March 2018.

First Author, Year, Country	Study Design Regional Description	Years of Outcome Ascertainment	Exposure Description	Pregnancy Outcome	Summary of Findings
Albouy-Llaty, 2016 France [107]	Historic cohort study Deux-Sèvres	2005–2010	Measurements of atrazine metabolites and NO_3_ in community water systems (263 municipalities) were linked to birth addresses	Preterm birth	No association for >26.99 mg/L vs. <14.13 mg/L NO_3_ in community water systems with or without atrazine detections, adjusted for neighborhood deprivation
Brender, 2013 Weyer, 2014 USA [38]	Population-based case-control study Iowa and Texas	1997–2005	Maternal addresses during the first trimester linked to public water utility nitrate measurements; nitrate intake from bottled water estimated with survey and laboratory testing; nitrate from private wells predicted through modeling; nitrate ingestion (NO_3_) estimated from reported water consumption	Congenital heart defects Limb deficiencies Neural tube defects Oral cleft defects	≥5 vs. <0.91 mg/day NO_3_ from drinking water spina bifida OR = 2.0 (95% CI: 1.3, 3.2) ≥5.42 vs. <1.0 mg/day NO_3_ from water: limb deficiencies OR = 1.8 (CI: 1.1, 3.1); cleft palate OR = 1.9 (CI: 1.2, 3.1) cleft lip OR = 1.8 (CI: 1.1, 3.1)
Holtby, 2014 Canada [113]	Population-based case-control study Kings County, Nova Scotia	1988–2006	Maternal addresses at delivery linked to municipal water supply median nitrate (NO_3_-N) concentrations; nitrate in rural private wells estimated from historic sampling and kriging	Congenital malformations combined into one group	Conceptions in 1987–1997: no association with nitrate concentrations Conceptions in 1998–2006: 1–5.56 mg/L NO_3_-N (vs. <1 mg/L) OR = 2.44 (CI: 1.05, 5.66); ≥5.56 mg/L OR = 2.25 (CI: 0.92, 5.52)
Joyce, 2008 Australia [109]	Record-based prevalence study Perth	2002–2004	Linked birth residences to 24 water distribution zones; computed average NO_3_-N mg/L from historical measurements; independent sampling conducted for 6 zones as part of exposure validation; also evaluated trihalomethanes (THM)	Premature rupture of membranes at term (PROM) (37 weeks’ gestation or later)	ORs for tertiles (vs. <0.125 mg/L NO_3_-N): 0.125–0.350 mg/L OR = 1.23 (CI: 1.03, 1.52); >0.350 mg/L OR = 1.47 (CI: 1.20, 1.79) No association with THM levels
Mattix, 2007 USA [110]	Ecologic study Indiana	1990–2002	Monthly abdominal wall defect rates linked to monthly surface water nitrate and atrazine concentrations (USGS-NAWQA monitoring data ^b^)	Abdominal wall birth defects	No correlation observed between nitrate levels in surface water and monthly abdominal wall defects Positive correlation with atrazine levels
Migeot, 2013 France [26]	Historic cohort study Deux-Sèvres	2005–2009	Measurements of atrazine metabolites and NO_3_ in community water systems (263 municipalities) were linked to birth addresses	Small-for-gestational age (SGA) births	ORs for tertiles (vs. <14.13 mg/L NO_3_) in community water systems with no atrazine detections: 14–27 mg/L OR = 1.74 (CI: 1.10, 2.75); >27 mg/L OR = OR 1.51 (CI: 0.96, 2.4); no association with nitrate when atrazine was detected
Stayner, 2017 USA [108]	Ecologic study 46 counties in Indiana, Iowa, Missouri, and Ohio	2004–2008	Counties had one or more water utility in EPA’s atrazine monitoring program; excluded counties with >20% of population on private wells and >300,000 population. Computed county-specific monthly weighted averages of NO_3_-N in finished drinking water; exposure metric was average 9 months prior to birth	Preterm birth Low birth weight	Average nitrate not associated with low birth weight and preterm birth Very low birth weight: RR for 1 ppm increase in NO_3_-N = 1.17 (CI: 1.08, 1.25); Very preterm birth RR for 1 ppm increase = 1.08 (CI: 1.02, 1.15)
Waller, 2010 USA [111]	Population-based case-control study Washington State	1987–2006	Calculated distance between maternal residence and closest stream monitoring site with concentrations >MCL for NO_3_-N, NO_2_-N, or atrazine in surface water (USGS-NAWQA data ^b^)	Gastroschisis	Gastroschisis was not associated with maternal residential proximity to surface water with elevated nitrate (>10 mg/L) or nitrite (>1 mg/L)
Winchester, 2009 USA [112]	Ecologic study USA-wide	1996–2002	Rates of combined and specific birth defects (computed by month of last menstrual period) linked to monthly surface water nitrate concentrations (USGS-NAWQA data ^b^); also evaluated atrazine and other pesticides (combined)	Birth defects categorized into 22 groups	Birth defect category “other congenital anomalies”: OR for continuous log nitrate = 1.15 (CI: 1.12, 1.18); adjusted for atrazine and other pesticides: OR = 1.18, CI: 1.14, 1.21); No association with other birth defects

Abbreviations: CI, 95% CI confidence interval; OR, odds ratio; RR, rate ratio; USGS-NAWQA, U. S. Geological Survey National Water Quality Assessment; ^a^ nitrate units are specified as reported in publications. NO_3_ can be converted to NO_3_-N by multiplying by 0.2258; ^b^ USGS-NAWQA data for 186 streams in 51 hydrological study areas; streams were not drinking water sources.

**Table 2:** Case-control and cohort studies of drinking water nitrate and cancer (January 2004–March 2018) by cancer site.

First Author (Year) Country	Study Design, Years Regional Description	Exposure Description	Cancer Sites Included	Summary of Drinking-Water Findings ^a,b^	Evaluation of Effect Modification ^c^
Zeegers, 2006 Netherlands [131]	Cohort Incidence, 1986–1995 204 municipal registries across the Netherlands	1986 nitrate level in 364 pumping stations, exposure data available for 871 cases, 4359 members of the subcohort	Bladder	Highest vs. lowest quintile intake from water (≥1.7 mg/day NO_3_-N [median 2.4 mg/day] vs. <0.20) RR = 1.11 (CI: 0.87–1.41; *p*-trend = 0.14)	No interaction with vitamin C, E, smoking
Espejo-Herrera, 2015 Spain [33]	Hospital-based multi-center case-control Incidence, 1998–2001 Asturias, Alicante, Barcelona, Vallès-Bages, Tenerife provinces	Nitrate levels in PWS (1979–2010) and bottled water (measurements of brands with highest consumption based on a Spanish survey); analyses limited to those with ≥70% of residential history with nitrate estimate (531 cases, 556 controls)	Bladder	Highest vs. lowest quartile average level (age 18-interview) (≥2.26 vs. 1.13 mg/L NO_3_-N) OR = 1.04 (CI: 0.60–1.81) Years >2.15 mg/L NO_3_-N (75th percentile) (>20 vs. 0 years) OR = 1.41 (CI: 0.89–2.24)	No interaction with vitamin C, E, red meat, processed meat, average THM level
Jones, 2016 USA [31]	Population-based cohort of postmenopausal women ages 55–69 Incidence, 1986–2010 Iowa	Nitrate levels in PWS (1955–1988) and private well use among women >10 years at enrollment residence with nitrate and trihalomethane estimates (20,945 women; 170 bladder cases); no measurements for private wells Adjusted for total trihalomethanes (TTHM)	Bladder	Highest vs. lowest quartile PWS average (≥2.98 vs. <0.47 mg/L NO_3_-N) HR = 1.47 (CI: 0.91–2.38; *p*-trend = 0.11) Years >5 mg/L (≥4 years vs. 0) HR = 1.61 (CI: 1.05–2.47; *p*-trend = 0.03) Private well users (vs. <0.47 mg/L NO_3_-N on PWS) HR = 1.53 (CI: 0.93–2.54)	Interaction with smoking (*p*-interaction = 0.03); HR = 3.67 (CI: 1.43–9.38) among current smokers/≥2.98 mg/L vs. non-smokers/<0.47 mg/L NO_3_-N); No interaction with vitamin C, TTHM levels
Mueller, 2004 USA, Canada, France, Italy, Spain [139]	Pooled case-control studies Incidence among children <15 years (USA <20 years) 7 regions of 5 countries	Water source during pregnancy and first year of child’s life (836 cases, 1485 controls); nitrate test strip measurements of nitrate and nitrite for pregnancy home (except Italy) (283 cases, 537 controls; excluding bottled water users: 207 cases, 400 controls)	Brain, childhood	Private well use versus PWS associated with increased risk in 2 regions and decreased risk in one; No association with nitrate levels in water supplies Astrocytomas (excludes bottled water users): ≥1.5 vs. <0.3 mg/L NO_2_-N OR = 5.7 (CI: 1.2–27.2)	Not described
Brody, 2006 USA [137]	Case-control Incidence, 1988–1995 Cape Cod, Massachusetts	Nitrate levels in public water supplies (PWS) since 1972 was used as an indicator of wastewater contamination and potential mammary carcinogens and endocrine disrupting compounds; excluded women on private wells	Breast	Average ≥1.2 mg/L NO_3_-N vs. <0.3 OR = 1.8, (CI: 0.6–5.0); summed annual NO_3_-N ≥ 10 vs. 1–< 10 mg/L OR = 0.9, CI: 0.6–1.5); number of years >1 mg/L NO_3_-N ≥8 vs. 0 years OR = 0.9 (CI: 0.5–1.5)	Not described
Inoue-Choi, 2012 USA [128]	Population-based cohort of postmenopausal women ages 55–69 Incidence, 1986–2008 Iowa	Nitrate levels in PWS (1955–1988) and private well use among women >10 years at enrollment residence (20,147 women; 1751 breast cases); no measurements for private wells	Breast	Highest vs. lowest quintile PWS average (≥3.8 vs. ≤0.32 mg/L NO_3_-N) HR = 1.14 (CI: 0.95–1.36; *p*-trend = 0.11); Private well (vs. ≤ 0.32 mg/L NO_3_-N) HR = 1.14 (CI: 0.97–1.34); Private well (vs. ≤0.32 mg/L NO_3_-N on PWS) HR = 1.38 (CI: 1.05–1.82); No association among those with low folate <400 µg/day	Interaction with folate for PWS (*p*-interaction = 0.06). Folate ≥400 µg/d: (≥3.8 vs. ≤0.32 mg/L NO_3_-N) HR = 1.40 (CI: 1.05–1.87; *p*-trend = 0.04)
Espejo-Herrera, 2016 Spain [138]	Hospital-based multi-center case-control Incidence, 2008–2013 Spain (8 provinces)	Nitrate levels in PWS (2004–2010), bottled water measurements and private wells and springs (2013 measurements in 21 municipalities in León, Spain, the area with highest non-PWS use) Analyses include women with ≥70% of period from age 18 to 2 years before interview (1245 cases, 1520 controls)	Breast	Water nitrate intake based on average nitrate levels (age 18 to 2 years prior to interview) and water intake (L/day). Post-menopausal women: >2.0 vs. 0.5 mg/day NO_3_-N OR = 1.32 (0.93–1.86); Premenopausal women: >1.4 vs. 0.4 mg/day NO_3_-N OR = 1.14 (0.67–1.94)	No interaction with red meat, processed meat, vitamin C, E, smoking for pre- and post-menopausal women
McElroy, 2008 USA [134]	Population-based case-control, women Incidence, 1990–1992 and 1999–2001 Wisconsin	Limited to women in rural areas with no public water system (475 cases, 1447 controls); nitrate levels at residence (presumed to be private wells) estimated by kriging using data from a 1994 representative sample of 289 private wells	Colorectal	All colon cancers: Private wells ≥10.0 mg/L NO_3_-N vs. <0.5 OR = 1.52 (CI: 0.95–2.44); Proximal colon cancer: OR = 2.91 (CI: 1.52–5.56)	Not described
Espejo-Herrera, 2016 Spain, Italy [135]	Multi-center case-control study Incidence, 2008–2013 Spain (9 provinces) and population-based controls; Italy (two provinces) and hospital-based controls	Nitrate levels in PWS (2004–2010) for 349 water supply zones, bottled water (measured brands with highest consumption), and private wells and springs (measurements in 2013 in 21 municipalities in León, Spain, the area with highest non-PWS use) Analyses include those with nitrate estimates for ≥70% of period 30 years before interview (1869 cases, 3530 controls)	Colorectal	Water nitrate intake based on average nitrate levels (estimated 30 to 2 years prior to interview) and water intake (L/day) Highest vs. lowest exposure quintiles (≥2.3 vs. <1.1 mg /day NO_3_-N) OR = 1.49 (CI:1.24–1.78); Colon OR = 1.52 (CI: 1.24–1.86), Rectum OR = 1.62 (CI: 1.23–2.14)	Interaction with fiber for rectum (*p*-interaction = 0.07); >20 g/day fiber + >1.0 mg/L NO_3_-N vs. <20 g/day + ≤1.0 mg/L HR = 0.72 (CI: 0.52–1.00). No interaction with red meat, vitamin C, E
Fathmawati, 2017 Indonesia [136]	Hospital-based case-control Incidence, 2014–2016 Indonesia (3 provinces)	Nitrate levels in well water collected during the raining season (Feb-March 2016) and classified based on >11.3 or ≤11.3 mg/L as NO_3_-N and duration of exposure >10 and ≤10 years Analyses included participants who reported drinking well water (75 cases, 75 controls)	Colorectal	Water nitrate > WHO standard vs. below (> 11.3 vs. ≤11.3 mg/L NO_3_-N) OR = 2.82 (CI: 1.08–7.40); > 10 years: 4.31 (CI: 11.32–14.10); ≤10 years: 1.41 (CI: 0.14–13.68)	Not described
Schullehner, 2018 Denmark [32]	Population-based record-linkage cohort of men and women ages 35 and older, 1978–2011 Denmark	Nitrate levels in PWS and private wells among 1,742,321 who met exposure assessment criteria (5944 colorectal cancer cases, including 3700 with colon and 2308 with rectal cancer)	Colorectal	Annual average nitrate exposure between ages 20–35 among those who lived ≥75% of study period at homes with a water sample within 1 year (61% of Danish population). Highest vs. lowest exposure quintile (≥2.1 vs. 0.16 mg/L NO_3_-N); Colorectal: HR = 1.16 (CI: 1.08–1.25); colon: 1.15 (CI: 1.05–1.26); rectum: 1.17 (CI: 1.04–1.32)	No information on dietary intakes or smoking
Ward, 2007 USA [34]	Population-based case control Incidence, 1986–1989 Iowa	Nitrate levels in PWS among those with nitrate estimates for ≥70% of person-years ≥1960 (201 cases, 1244 controls)	Kidney (renal cell carcinomas)	Highest vs. lowest quartile PWS average (≥2.8 mg/L NO_3_-N vs. <0.62) OR = 0.89 (CI 0.57–1.39); Years >5mg/L NO_3_-N 11+ vs. 0 OR = 1.03 (CI: 0.66–1.60)	Interaction with red meat intake (*p*-interaction = 0.01); OR = 1.91 (CI 1.04–3.51) among 11+ years >5 mg/L NO_3_-N and red meat ≥1.2 servings/day. Interaction with vitamin C showed similar pattern (*p*-interaction = 0.13)
Jones, 2017 USA [127]	Population-based cohort of postmenopausal women ages 55–69 Incidence, 1986–2010 Iowa	Nitrate levels in PWS (1955–1988) and private well use among women >10 years at enrollment residence. PWS measurements for nitrate and TTHM; no measurements for private wells (20,945 women; 163 kidney cases)	Kidney	Nitrate and TTHM metrics computed for duration at water source (11+ years) 95th percentile vs. lowest quartile PWS average (≥5.00 vs. <0.47 mg/L NO_3_-N) HR = 2.23 (CI: 1.19–4.17; *p*-trend = 0.35) Years >5 mg/L (≥4 years vs. 0) HR = 1.54 (CI: 0.97–2.44; *p*-trend = 0.09) Private well users (vs. <0.47 mg/L NO_3_-N in PWS) HR = 0.96 (CI: 0.59–1.58)	No interaction with smoking, vitamin C
Ward, 2006 USA [35]	Population-based case-control Incidence, 1998–2000 Iowa	Nitrate levels in PWS among those with nitrate estimates for ≥70% of person-years ≥1960 (181 case, 142 controls); nitrate measurements for private well users at time of interviews (1998–2000; 54 cases, 44 controls)	Non-Hodgkin lymphoma	Private wells: >5.0 mg/L NO_3_-N vs. ND OR = 0.8 (CI 0.2–2.5) PWS average: ≥2.9 mg/L NO_3_-N vs. <0.63 OR = 1.2 (CI 0.6–2.2) Years ≥5mg/L NO3-N: 10+ vs. 0 OR = 1.4 (CI: 0.7–2.9)	No interaction with vitamin C, smoking
Inoue-Choi, 2015 USA [129]	Population-based cohort of postmenopausal women ages 55–69 Incidence, 1986–2010 Iowa	Nitrate levels in PWS (1955–1988) and private well use among women >10 years at enrollment residence; PWS measurements for nitrate and TTHM; no measurements for private wells (17,216 women; 190 ovarian cases)	Ovary	Nitrate and TTHM metrics computed for reported duration at water source (11+ years) Highest vs. lowest quartile PWS average (≥2.98 mg/L vs. <0.47 mg/L NO_3_-N) HR = 2.03 (CI = 1.22–3.38; *p*-trend = 0.003) Years >5 mg/L (≥4 years vs. 0) HR = 1.52 (CI: 1.00–2.31; *p*-trend = 0.05) Private well users (vs. <0.47 mg/L NO_3_-N in PWS) HR = 1.53 (CI: 0.93–2.54)	No interaction with vitamin C, red meat intake, smoking for PWS nitrate Interaction with private well use and vitamin C intake (*p*-interaction = 0.01)
Quist, 2018 USA [126]	Population-based cohort of postmenopausal women ages 55–69 Incidence, 1986–2011 Iowa	Nitrate levels in PWS (1955–1988) and private well use among women >10 years at enrollment residence; nitrate and TTHM estimates for PWS (20,945 women; 189 pancreas cases); no measurements for private wells Adjusted for TTHM (1955–1988), measured levels in 1980s, prior year levels estimated by expert)	Pancreas	Nitrate and TTHM metrics computed for reported duration at water source (11+ years) 95th percentile vs. lowest quartile PWS average (≥5.69 vs. <0.47 mg/L NO_3_-N) HR = 1.16 (CI: 0.51–2.64; *p*-trend = 0.97) Years >5 mg/L (≥4 years vs. 0) HR = 0.90 (CI: 0.55–1.48; *p*-trend = 0.62) Private well users (vs. <0.47 mg/L NO_3_-N) HR = 0.92 (CI: 0.55–1.52)	No interaction with smoking, vitamin C
Ward, 2008 USA [133]	Population-based case control Incidence, 1988–1993 Nebraska	Controls from prior study of lymphohematopoetic cases and controls interviewed in 1992–1994; Proxy interviews for 80%, 76%, 61% of stomach, esophagus, controls, respectively. Nitrate levels (1965–1985) in PWS for ≥70% of person-years (79 distal stomach, 84, esophagus, 321 controls); Private well users sampling at interview (15 stomach, 22 esophagus, 44 controls)	Stomach and esophagus (adenocarcinomas)	Highest vs. lowest quartile PWS average (>4.32 vs. <2.45 mg/L NO_3_-N): stomach OR = 1.2 (CI 0.5–2.7); esophagus OR = 1.3 (CI: 0.6–3.1); Years >10 mg/L NO_3_-N (9+ vs. 0): stomach OR = 1.1 (CI: 0.5–2.3); esophagus OR = 1.2 (CI: 0.6–2.7) Private well users (>4.5 mg/L NO_3_-N vs. <0.5) stomach OR = 5.1 (CI: 0.5–52; 4 cases, 13 controls); esophagus OR = 0.5 (CI: 0.1–2.9; 8 cases; 13 controls)	No interaction with vitamin C, processed meat, or red meat for either cancer
Ward, 2010 USA [37]	Population-based cohort of postmenopausal women ages 55–69 Incidence, 1986–2004 Iowa	Nitrate levels in PWS (1955–1988) and private well use among women >10 years at enrollment residence (21,977 women; 40 thyroid cases); no measurements for private wells	Thyroid	Highest vs. lowest quartile PWS average (>2.46 vs. <0.36 mg/L NO_3_-N) HR = 2.18 (CI: 0.83–5.76; *p*-trend = 0.02) Years >5 mg/L (≥5 years vs. 0) HR = 2.59 (CI: 1.09–6.19; *p*-trend = 0.04); Private well (vs. <0.36 mg/L NO_3_-N on PWS) HR = 1.13 (CI: 0.83–3.66) Dietary nitrate intake quartiles positively associated with risk (*p*-trend = 0.05)	No interaction with smoking, vitamin C, body mass index, education, residence location (farm/rural vs. urban)

ND = not detected; PWS = public water supplies; ^a^ nitrate or nitrite levels presented in the publications as mg/L of the ion were converted to mg/L as NO_3_-N or NO_2_-N; ^b^ Odds ratios (OR) for case-control studies, incidence rate ratios (RR) and hazard ratios (HR) for cohort studies, and 95% confidence intervals (CI); ^c^ Factors evaluated are noted. Interaction refers to reported *p* ≤ 0.10 from test of heterogeneity.
